# Cytotoxicity of *Aconitum* alkaloid and its interaction with calf thymus DNA by multi-spectroscopic techniques

**DOI:** 10.1038/s41598-017-15240-9

**Published:** 2017-11-06

**Authors:** Fei Liu, Xiaoxin Tan, Xu Han, Xiang Li, Nan Li, Weijun Kang

**Affiliations:** 1 School of Basic Medical, Hebei University of Chinese Medicine, Shijiazhuang, Hebei Province China; 2 School of Public Health, Hebei Medical University, Shijiazhuang, Hebei Province China; 3 Institute of Viral Disease, Hebei Center for Disease Control and Prevention, Shijiazhuang, Hebei Province China

## Abstract

The cytotoxicities of three aconitum alkaloids- aconitine, hypaconitine and mesaconitine, and their abilities to bind DNA have been explored. Rat myocardial cells H9c2 were treated with aconitum alkaloids and assessed the cytotoxicities by using MTT assay and flow cytometry. Apoptosis was evidenced by the results of the annexin V/propidium iodide (PI) assay. Aconitine was found to be the most toxic in rat myocardial cells H9c2 in three aconitum alkaloids. At the same time, DNA adducts were isolated and then analyzed by UV-Vis spectroscopy after exposure to alkaloids, which indicated that three alkaloids could bind to DNA in rat myocardial cells H9c2. Furthermore, their binding modes were investigated by UV-Visible, fluorescence, DNA melting studies and ionic strength effect. Results indicated that the interaction between three alkaloids and DNA were intercalation coupled with electrostatic effect. The estimated binding constants were between 4.83 × 10^5^ M^−1^ to 9.85 × 10^5^ M^−1^ for three alkaloids at 298 K.

## Introduction

**Figure 1:**
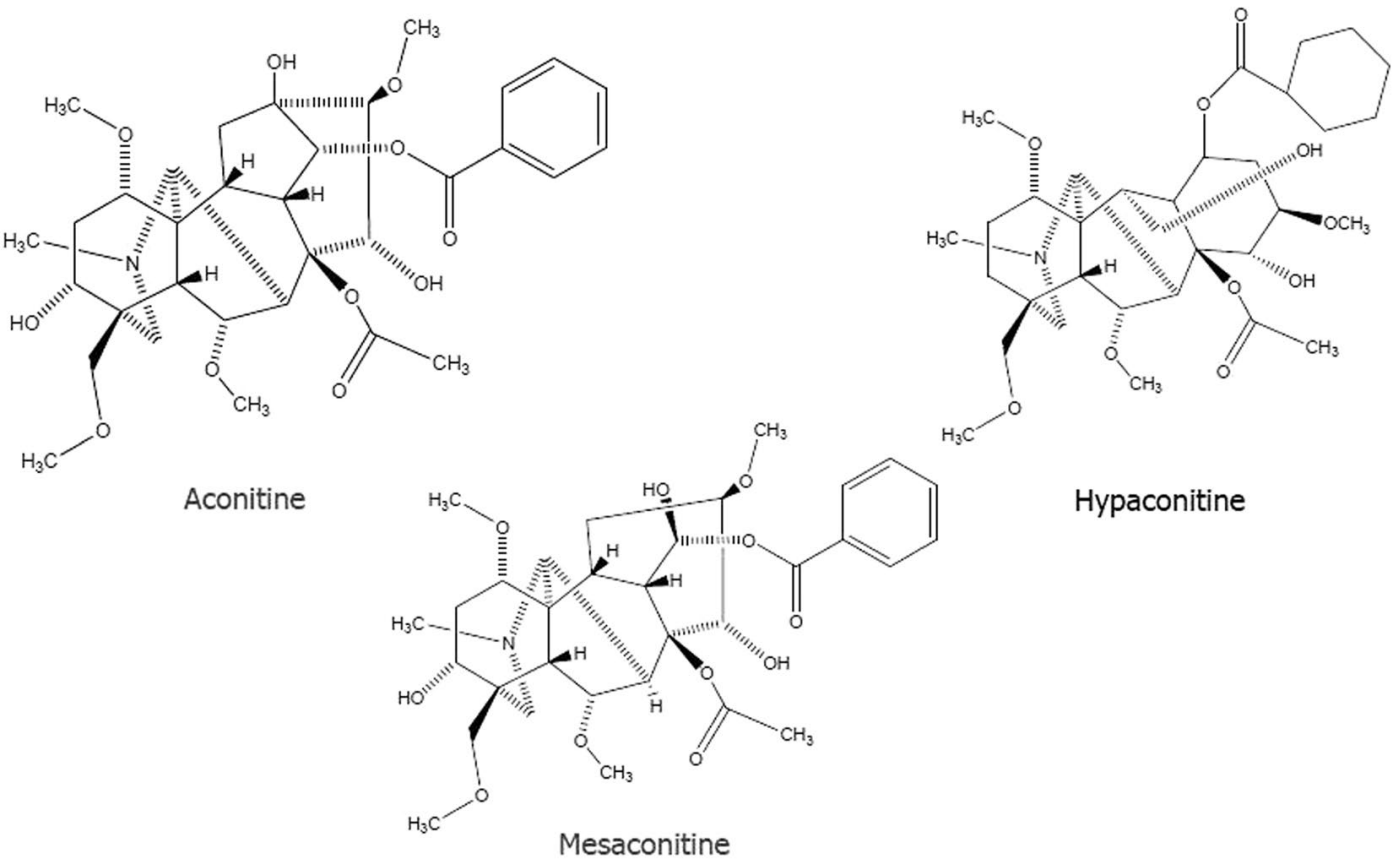
Structural formulas of aconitine, hypaconitine and mesaconine.

The *Aconitum* species (Ranumculaceae) including Aconitum carmichaeli Debx. and Aconitum Kusnezoffii Reichb. are widespread throughout Asia, Europe and North America. Aconite is usually applied for various diseases, such as painful joints, gastroenteritis, rheumatic fever, and various tumors^1^. *Aconitum* is a botanical source including various pharmaceutically active components. At least 224 alkaloids and some flavonoid glycosides in addition to β-sitosterol have been identified from various parts of the plant^1^. Diterpenoid alkaloid, which is one of the most important components in the plant, has been commonly used in traditional Chinese medicine (TCM) for thousands of years. However, unprocessed *Aconitum* is forbidden to sell directly on the market by the State Administration of Traditional Chinese Medicine in China due to its intense toxicity-which probably results in fatal cardiac poisoning. Cases of aconite poisoning have been reported from many countries especially China. Moreover, with the increasing recognition of alternative medicine, there were quite a few clinical cases of aconite intoxication reported in the United States and European countries^2–4^. Unprocessed *Aconitum* contains highly toxic alkaloids with a diester diterpene structure such as hypaconitine (HA), aconitine (AC), and mesaconitine (MA) (Fig. 1)^5^. The whole plant of aconite is highly toxic, with the concentration of diester-diterpene alkaloids (DDA) higher in flowers and roots than in stems and leaves. The intoxication patient may present the typical symptoms of AC poisoning after ingestion of the roots of the plant, such as vomiting, nausea, dizziness, hypotension, even coma, furthermore, death could occur from ventricular arrhythmia^1^.

So far as we know, different *Aconitum* sources contain varies alkaloid: MA is the main compound of *Aconitum kusnezoffii*, AC is the main alkaloid in *Aconitum napellus*, and the main constituents of *Aconitum carmichaeli* are HA and MA. Aconitine, which has a narrow therapeutic index, is considered to be the foremost highly toxic DDA in aconitum alkaloids^6^. Wada *et al*. reported that the LD_50_ values of aconitine were 1.8 mg/kg p.o., 0.12 mg/kg i.v., 0.31 mg/kg i.p.in mice^7^. Furthermore, for humans, the lethal dose of aconitine is estimated to be 1–2 mg for a healthy man with a body weight of 70 kg (15–30 μg/kg)^8^. Although the LD_50_ values of hypaconitine and mesaconitine were significantly higher than aconitine, three of the alkaloids are all held responsible for the toxic effects. Due to DDA’s hyperpolarization and activation effect on the voltage-dependent sodium channels of the nerves and myocardium, AC, MA and HA were served as highly toxic neurotoxins and cardiotoxins which resulted in a toxic symptoms consisting of neurological, gastrointestinal, and cardiovascular signs^9,10^.

The interaction of drug and DNA has long been the focus of research in chemistry and life science, especially important for the designing of new drug and DNA damage mechanism study. So far as we know, drug-DNA interaction is considered to be the reason for the DNA damage of some drugs and pollutants, such as ochratoxin A (OTA) and aristolochic acids (AAs)^11^. Although the toxic mechanism presented by most alkaloids remains unknown, many studies revealed that lots of alkaloids (e.g., evodiamine,vinblastine, and harmalol) had the ability to cleave or bind with DNA^12–14^. DNA is viewed as one of the main molecular targets for toxic effect of many alkaloids. Generally speaking, the interaction between DNA and drugs was investigated with Calf thymus DNA (CT-DNA) *in vitro*^15,16^; while little attention is focused on whether drug could really bind or cleave DNA *in vivo*. Thus, DNA-adducts need to be examined using UV-Visible spectroscopy after exposure to various concentrations of DDAs.

In recent years, there were still a few clinical cases of aconite intoxication reported from many countries^17–19^. Most cases of DDA poisoning were due to the mistaken ingestion of aconite as edible wild plants or improper consumption of AC-containing traditional herbal medicine^2,20^. The aim of the present study was to explore cytotoxicity and DNA-binding ability of DDAs (AC, MA and HA) in rat myocardial cells H9c2. Assessment of cytotoxicity was done by MTT assay and flow cytometry. Furthermore, the interaction between DNA and DDAs *in vitro* and *vivo* were studied by the application of UV-vis and fluorescence spectroscopy.

## Results

### Cytotoxicity of AC, MA and HA in rat myocardial cells H9c2

**Figure 2:**
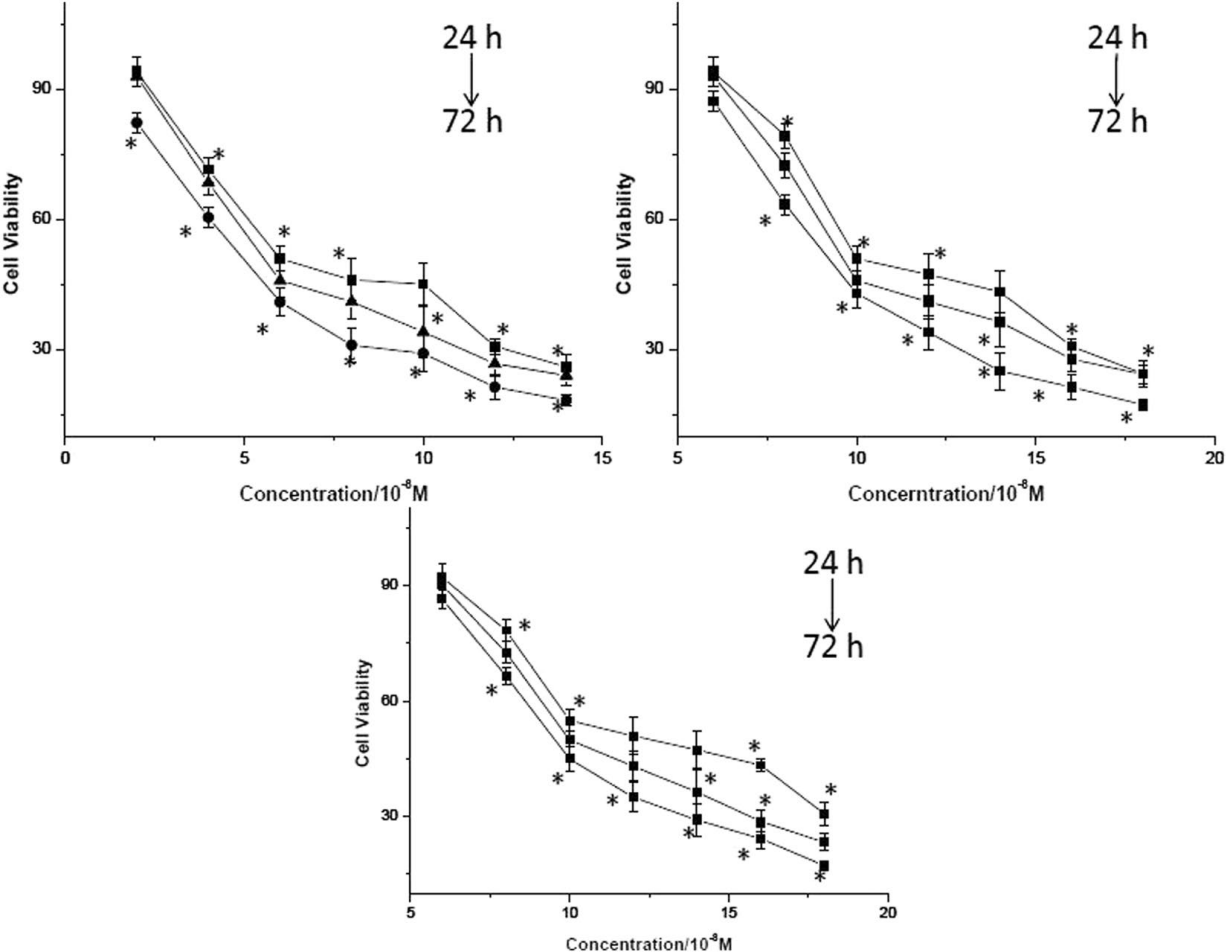
Cytotoxicity of AC (**A**), HA (**B**) and MA (**C**) in rat myocardial cells H9c2. *p < 0.05 represent significant difference with DMEM control.

The cytotoxicity of three alkaloids in rat myocardial cells H9c2 was determined using MTT assay. The cell viability results following incubation with various concentrations of AC, MA and HA for 24, 48 and 72 h were shown in Fig. 2 and IC_50_ values were provided in Table 1. Comparing with the control cells, rat myocardial cells H9c2 exposed to DDAs showed a significant dose- and time-dependent inhibition of cell growth (*P* < 0.05 vs. control). DMSO did not show any inhibitory effect on the cell viability.

**Table 1:** The median inhibition concentration (IC_50_) of AC, MA and HA in rat myocardial cells H9c2 following incubation for 24, 48 and 72 h, respectively.

Compounds	IC_50_(10^−8^ M)		
	24 h	48 h	72 h
AC	6.9 ± 0.31	6.1 ± 0.42	5.6 ± 0.89
MA	11.8 ± 0.88	10.6 ± 0.45	9.3 ± 0.45
HA	10.5 ± 0.78	11.2 ± 0.37	10.2 ± 0.87

Data represents mean ± 95% CI of three independent experiments.

### Morphology of rat myocardial cells H9c2 following treatment with AC, MA and HA

**Figure 3:**
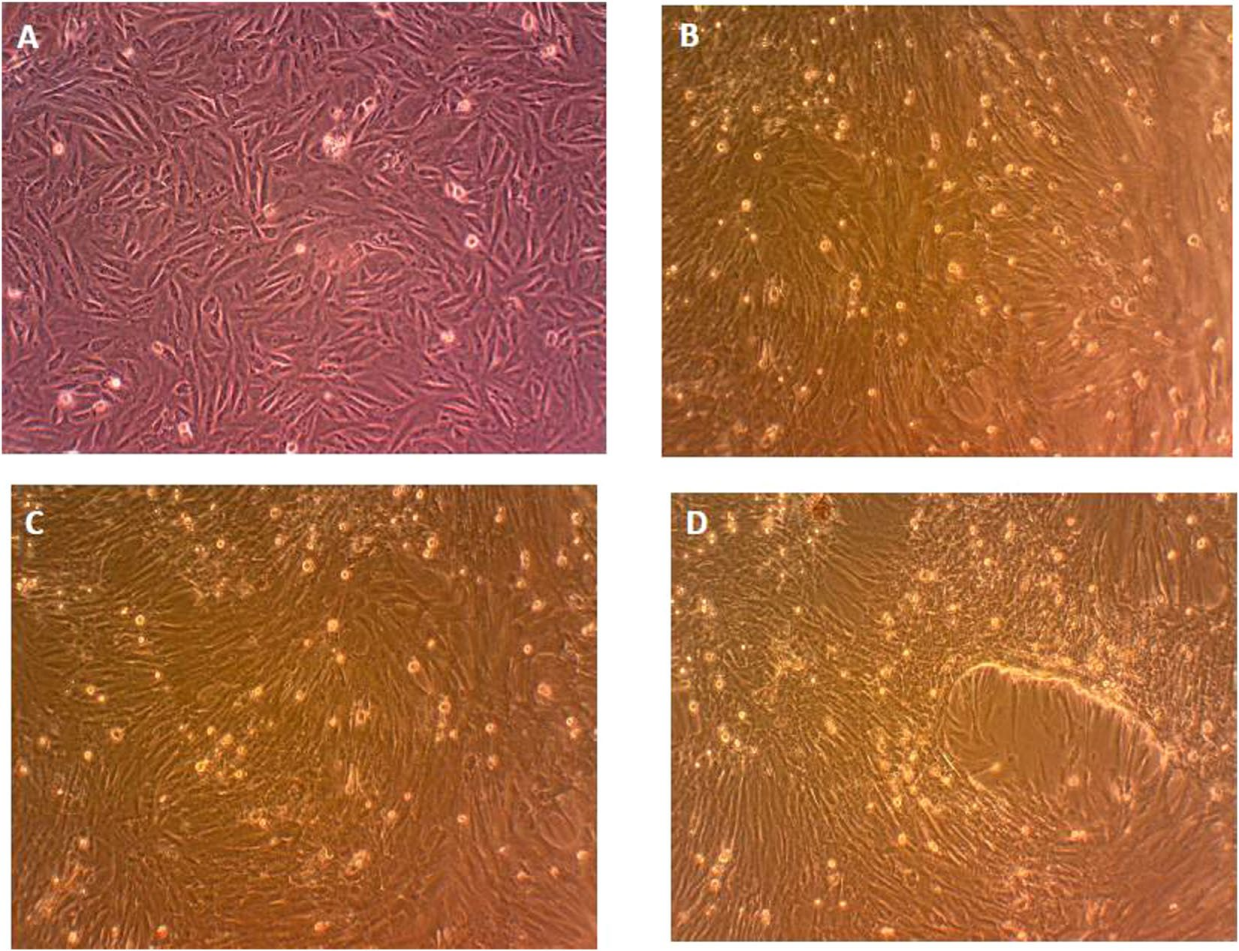
Morphology of rat myocardial cells H9c2 after exposure to DDAs for 24 h. (**A**) DMEM control; (**B**) AC; (**C**) HA; (**D**) MA.

A morphological change observed in rat myocardial cells H9c2 treated with DDAs was shown in Fig. 3. Cells without any treatment and cells treated with 0.5% DMSO (group of control) were egg and shuttle shaped; while cells treated with DDAs were shrinkage, irregularities, and cell rounding in contour and size. Rat myocardial cells H9c2 exposed to AC (6.9 × 10^−8^ M), MA (11.8 × 10^−8^ M) and HA (10.5 × 10^−8^ M) for 24 h showed similar morphology.

### Flow cytometric detection

**Figure 4:**
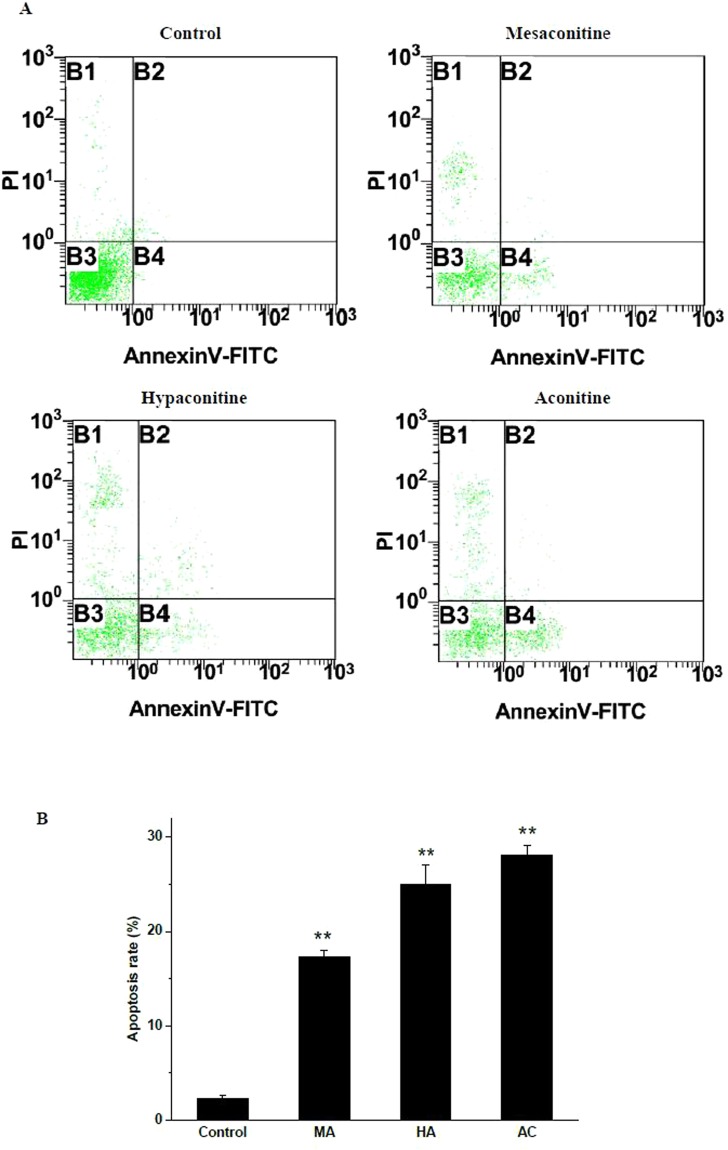
(**A**) Apoptosis induced by AC, HA and MA in H9c2 cells for 24 h. (**B**) The summary (**p < 0.05 vs. control group).

PI is used to distinguish necrotic cells from apoptotic and living cells by supravital staining without prior permeabilization. Annexin V, which has a high affinity for phosphatidylserine (PS), labeled with a fluorophore can identify apoptotic H9c2 by binding to PS. Early apoptotic cells tested positive for annexin V and negative for PI staining, whereas late apoptotic cells undergoing secondary necrosis were positive for both annexin V and PI staining. As shown in Fig. 4, all of the three alkaloids stimulated apoptosis in H9c2 cells without causing any obvious necrosis. In the control group, the percentage of apoptotic H9c2 cells was 2.3%; however, the percentage of apoptotic H9c2 increased up to 28.1%, 24.9% and 17.3% after treatment with aconitine, hypaconitine and mesaconitine, respectively.

### Absorption spectra of AC-DNA, HA-DNA and MA-DNA adducts

**Figure 5:**
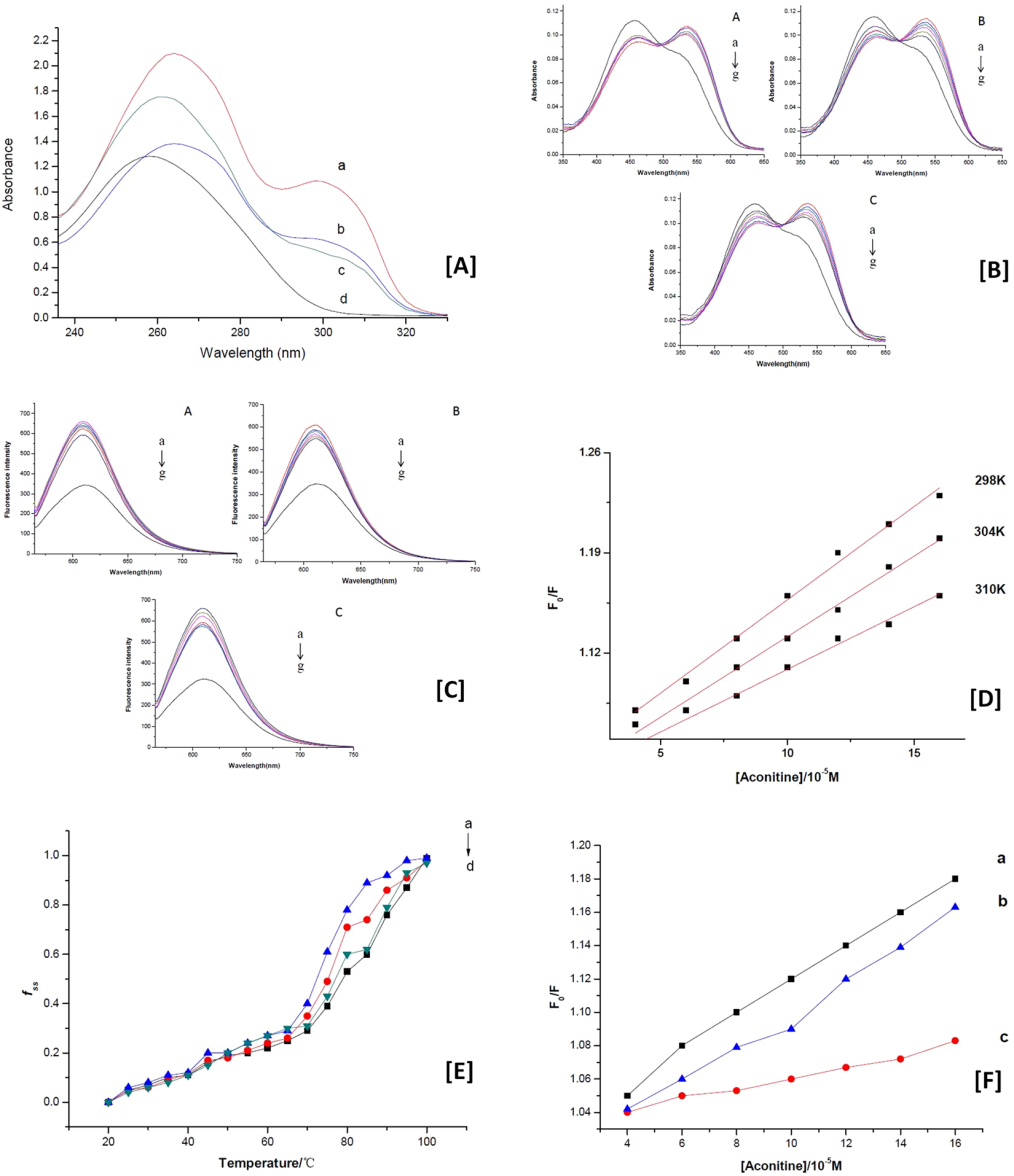
Absorption spectra and emission spectra of DNA interfered by DDAs. (**A**) Absorption spectra of DNA adduct following treatment with DDAs for 24 h. (**B**) Absorption spectra of DDAs interaction with CT-DNA. (**C**) Fluorescence quenching of NR-DNA complex by DDAs. (**D**) Stern-Volmer plots for aconitine at 298 K, 304 K and 310 K. (**E**) Tm value of DNA and DNA-DDA complex. (**F**) Influence of ionic strength on fluorescence quenching.

UV-Vis spectroscopic data collected from DNA, which was isolated from rat myocardial cells H9c2, is displayed in Fig. 5A (line a: cells treated with AC; line b: cells treated with HA; line c: cells treated with MA; line d: control). The absorption spectrum of DNA isolated from the cells in control group had a peak at 258 nm (line d), which was generated from the strong absorption of purine and pyrimidine bases in DNA. The ratio of the absorbance at 260 nm and 280 nm was greater than 1.8, indicating that the DNA was sufficiently free from protein^21^. After exposure to various concentrations of DDAs, the peak position of DNA had a slight redshift (from 258 nm to262 nm). The changes of spectra for DNA isolated from cells after treated with AC, HA, and MA were almost the same. Generally speaking, absorption spectroscopy is considered to be one of the most useful techniques in DNA-binding studies. Owing to the binding of drugs with DNA, the absorbance spectrum shows hyperchromism (or hypochromism) effect and a blueshfit (or redshift), which involve a strong stacking interaction between base pairs of DNA and the drugs^22,23^. The red shift of absorption spectra and the formation of the new absorption peak revealed the interaction between alkaloids and DNA bases. The results showed all of the three DDAs could bind into DNA *in vivo*. DDA-DNA adducts had formed through some of the binding modes.

### Absorption spectra of DDAs interaction with CT-DNA

In general, if a small molecule of drug interacts with DNA, changes in absorbance and in the position of the band should occur. Hypochromic effects and a bathochromic shift could be observed especially when drug interacts with DNA by either the electrostatic effect or the intercalation binding. As we know, neutral red (NR) is reported as a mutagenic intercalating dye for DNA^23^. In this work, with the gradual addition of DNA, the intensity band of NR at 454 nm decreased, and a strong shoulder at 543 nm developed. On this basis, a series of AC, MA and HA at different concentrations were added into the NR-DNA solution for further support of the binding mode between alkaloids and DNA. As shown in Fig. 5B, with the increasing concentration of DDAs, the maximum absorption at 543 nm of the NR-DNA spectrum decreased, meanwhile the absorption intensity in the developing band at 454 nm increased. Comparing with the spectrum of NR interaction with DNA, the result displayed the reverse process. Furthermore, there was no remarkable difference between experimental outcomes for AC, MA and HA. This revealed that NR molecule which has intercalated into the base pairs of DNA was replaced by DDAs, with the consequent outcome that the spectrum of the NR dye is nearly restored.

### Fluorescence quenching

In this work, none of the DDAs (AC, MA and HA) or DDA-DNA complex exhibited fluorescence in the buffer solution (Tris-HCl, pH = 7.4). Thus, molecule probe must be employed to explore the characteristics of fluorescence spectra of DDA-DNA interaction. As we know that neutral red is a kind of planar phenazine dye, which is structurally alike with other planar dyes, e.g., thiazine, acridine and xanthenes. Besides, upon addition of DDAs to a NR solution, neither significant change on fluorescence intensity for NR has been observed nor new fluorescence peaks developed. Based on this, NR was selected as the probe in this work, because of its lower toxicity and higher stability. The emission spectrum of the DNA-NR complex in the absence and presence of AC, MA and HA were shown in Fig. 5C, respectively. There was an obvious maximum emission at 612 nm when the DNA-NR system was excited at 546 nm. With an increasing sequentially adding of each DDA, the fluorescence intensity of the complex decreased without notable changes in the wavelength of maximum emission. Moreover, three of the alkaloids had the ability for fluorescence quenching of DNA-NR system. Figure 5C also showed a significant difference in quenching degree for three of the DDAs, which revealed the difference binding ability and binding constant (K_b_) for AC, MA and HA.

In this work, DDAs could not induce obvious change on the fluorescence intensity of NR, which means DDA and NR could not bind together. Considering UV-vis spectroscopic studies above, it seems that the decrease in the emission intensity on the addition of DDAs reflected the binding of AC, MA and HA to DNA by intercalation. The observation revealed that DDA competed against NR in binding with DNA and NR molecules intercalated in DNA double helix were excluded by alkaloids. Consequently, a decrease in the emission was observed. Thus, it can be deduced that the AC, MA and HA intercalated into DNA and formed the DDA-DNA complex.

In order to elucidate the fluorescence quenching mechanism, the classical Sterne-Volmer equation was utilized for data analysis.

$${F}_{0}/F=1+{K}_{q}{\tau }_{0}[D]=1+{K}_{{\rm{sv}}}[D]$$

*F*_0_ and *F* are the fluorescence intensities of DNA-NR complex with or without DDA. *K*_*sv*_ is the Stern-Volmer quenching constant; and τ_0_ is the average lifetime of the biomolecule without alkaloid (τ_0_ ≈ 10^−8^ s); and [*D*] is the DDA concentration^24^.

In general, the mechanisms of fluorescence quenching are classified as either dynamic or static quenching, which can be distinguished by their difference manifestations dependence on temperatures. Figure 5D showed the curves of *F*_0_/*F* versus [*D*] for aconitine at different temperatures (298, 304, and 310 K). For dynamic quenching, an increasing temperature means a larger quenching constant. As shown Fig. 5D, the values of *K*_*sv*_ decreased with the increasing temperatures. Furthermore, *K*_*q*_ (10^12^ L M^−1^ s^−1^) for all of the three DDAs were greater than the maximum diffusion constant of the biomacromolecules (2.0 × 10^10^ L mol^−1^ s^−1^), which confirmed that fluorescence quenching of DNA-NR complex by DDAs were static quenching.

Moreover, the binding stoichiometry (n) of the system and the apparent binding constant (*K*_*b*_) for alkaloids can be calculated by the equation below:

$${\rm{log}}\,[({F}_{0}\,-\,F)/F]=\,{\rm{log}}\,{K}_{b}+nlog[D]$$

*K*_*b*_ is the binding constant for DDA and n is the number of binding sites in DNA double helix. According to the equation, n is the slope of the plot for log [(*F*_*0*_ − *F*)/*F*] versus log [*D*]; and the results were summary in Table 2. The values of n obtained for DDAs were from 0.97 to 1.15, indicate the existence of just a single binding site in DNA for AC, MA and HA. Meanwhile, the results showed that AC bound to DNA with high affinity, which had a much higher binding constant than that of HA or MA, suggesting that the biological activities of AC were superior in three of the DDAs.

**Table 2:** Fluorescence quenching results for AC, HA and MA at 298 K.

DDA	*K*_*sv*_ (×10^4^ M^−1^)	*K*_*b*_ (×10^5^ M^−1^)	n	*R* ^a^
AC	8.92	9.85	1.04	0.994
HA	4.26	5.36	0.97	0.993
MA	4.18	4.83	1.15	0.990

^a^*R* is correlation coefficient.

### Melting studies

A DNA melting analysis is another strong evidence for drug intercalates into the base pairs of DNA. Interaction of small molecules with DNA can influence melting temperature (Tm) of DNA significantly. The intercalation binding of drug into double helix could stabilize the structure of DNA and Tm increases by about 5–8 °C, meanwhile, the other binding modes causes no obvious increase in Tm^25^.

Experiments were carried out by controlling various temperatures ever 5 °C from 20 to 100 °C in the absence and the presence of DDAs while monitoring the absorbance of DNA at 260 nm. Figure 5E showed the Tm value of CT-DNA was 74.1 °C under our experimental conditions, however it was increased to 79.2 °C, 80.1 °C and 81.7 °C in the presence of AC, HA and MA respectively. The results suggested that DDAs molecules intercalated into the base pairs and stabilized DNA helix. It is in a good agreement with the absorption spectra and fluorescence quenching study above.

### Effects of ionic strength

The effects of sodium chloride on the fluorescence quenching of AC, MA and HA were studied respectively, and the results were shown as Fig. 5F. Generally, there are three predominant non-covalent modes for small molecules binding to DNA, including intercalation, electrostatic interaction and groove binding. If the electrostatic binding is one of the interaction modes, with the adding of NaCl, small molecules of the alkaloids will release^22^; because Na^+^ ions can exhibit a tendency to bind with the phosphate groups of CT-DNA by electrostatic interaction, with a result of the binding between small molecule with DNA being much weaker.

As shown in Fig. 5F, with the addition of increasing concentrations of NaCl (0.1 M for line a, 0.2 M for line b, 0.3 M for line c), the fluorescence quenching by DDA for DNA-NR complex was decrease. The results indicated that besides intercalation, electrostatic binding was another reasonable binding mode between DDAs and DNA.

## Discussion

Many studies have demonstrated that diterpenoid alkaloids often had damage to tumor cells and normal cells with their cytotoxicity that interfere the DNA synthesis during the cell divisions. Several C19-norditerpenoids like neoline, aconitine, pubescenine, 14-deacetylajadine, lycoctonine, dehydrotakaosamine and ajadelphinine had irreversible effects on SkMel25, SW480, HeLa and PC12 cell lines^26,27^. As it’s known to all, cardiomyocyte damage is one of the most important factors for DDAs’s cardiotoxity. In our study, the *in vitro* cytotoxic activities of AC, MA and HA in rat myocardial cells H9c2 were examined using a cell viability assay. The experimental results clearly confirmed that AC, HA and MA could significantly inhibit the growth of the rat myocardial cells H9c2. DDAs at concentrations above 5 × 10^−8^ M were cytotoxic. The IC_50_ values by the MTT assay for AC, HA and MA were ranged from 6.9 × 10^−8^ M to 11.8 × 10^−8^ M after 24 h. The results from C.E. Ellis^28^ have shown that pavetamine, which induced cardiomyopathy of domestic ruminants, was cytotoxic in H9c2 cells at a concentration of 200 μM after exposure for 72 h. Cytotoxicity of piperazine derived drugs in the H9c2 rat cardiac cell were also investigated^29^. The IC_50_ values were 343.9, 570.1, and 702.5 μM for N-benzylpiperazine (BZP), 1-(4-methoxyphenyl) piperazine (MeOPP), and 1-(3,4-methylenedioxybenzyl) piperazine (MDBP), respectively.

Among the C19-diterpenoid alkaloids in our study, aconitine exhibited the strongest cytotoxic activity against the cells. Some case reports of *Aconitum* poisoning also illustrated that aconitine was a possible reason for fatal cardiac poisoning^30–32^. Metabolic profiles were showed that the number of metabolites in the AC and MA groups were dissimilar, with significant changes or with a tendency to change, suggested a possible difference in the toxicity mechanisms of these diterpenoid alkaloids^33^. Apoptosis has an essential function in the pathogenesis of cardiovascular diseases and contributes to the development of cardiovascular disorders^34^. In the present study, we used flow cytometry to identify apoptosis in H9c2 cells, and found that all of the three DDAs promoted the apoptotic response of heart *in vitro*. The apoptotic activity of aconitine was more potent and obvious than that of hypaconitine and mesaconitine. Our results were in a good agreement with the previous study which suggested aconitine could take the most responsibility for cardiac toxicity.

It is known that drug-DNA interaction is considered to be the reason for the DNA damage of some drugs^35^. Recent study reported that many alkaloids like sempervirine, harmalol, and evodiamine interacted with DNA by intercalation mode. Intercalation was also shown to be the main binding mode of vinblastine to DNA double helix, with a binding constant of 1.7 × 10^3^ M^−1 12^. It was reported that harmalol bind with DNA by intercalation with the estimated *K*_*b*_ of 4.5 × 10^5^ M^−1 13^. As far as we know, the reported binding constants for alkaloids binding to the base pairs of DNA were range from 10^3^ to 10^7^ M^−1^ ^12,13,36,37^. All the results in present study revealed that AC, HA and MA could interact with DNA *in vivo*. Binding of these three DDAs to CT-DNA *in vitro* were shown to be through the same mode; and similar to most alkaloids, intercalation and electrostatic interaction were the main binding mode between those DDAs and DNA. The estimated binding constants were 9.85 × 10^5^ M^−1^ for AC, 4.83 × 10^5^ M^−1^ for MA and 5.36 × 10^5^ M^−1^ for HA at 298 K, respectively. As shown that binding constant of AC is higher than those of MA and HA, which suggested the interaction between AC and DNA is stronger than other DDAs. There is a certain degree of correlation between the cardiac toxicity and DNA injury that caused by aconitine.

## Materials and Methods

### Materials

Rat myocardial cells H9c2 were obtained from Shanghai Institutes for Biological Sciences (Shanghai, China). AC, MA and HA were provided by Chengdu Must Bio-Technology Co., LTD (Chengdu, China). Trypsin/ethylene diaminetetraacetic acid (EDTA), fetal bovine serum (FBS) and Dimethyl sulfoxide (DMSO) were obtained from Gibco (NY, USA). Glutamine Minimum essential medium (DMEM) (99.9%), NR and Calf thymus DNA (CT-DNA) were obtained from Sigma (MO, USA).

CT-DNA was dissolved in 50 mM Tris-HCl buffer (pH 7.4) (including 150 mM NaCl). The concentration of stock solution for DNA was calculated to be 2 mM by absorption at 260 nm, following Beer-Lambert Law, using the molar absorption coefficient ε_260_ = 6600 L mol^−1^cm ^−1^ ^38^.

The stock solutions were stored at 4 °C for more than 24 h to obtain homogeneity.

### Cell culture and treatment

Rat myocardial cells H9c2 were maintained in DMEM supplemented with 1% glutamine, 10% FBS, 100 U/mL penicillin and 100 mg/mL streptomycin in an atmosphere at 37 °C humidified with 5% CO_2_. Stock solutions of DDAs were prepared in DMSO. Final concentration of test DDAs were achieved by adding the culture medium. Cells were treated with six dilutions of each individual DDA: AC (2.0~12.0 × 10^−8^ M), HA (6.0~14.0 × 10^−8^ M) and MA (6.0~14.0 × 10^−8^ M) at 24, 48 and 72 h of exposure. A solvent control and untreated cells were also used to evaluate the effect of DMSO on rat myocardial cells H9c2.

### MTT assay

Rat myocardial cells H9c2 were seeded at 10^3^–10^4^ cells/mL in 96-well plates. Twenty-four hours after planting, the cells were exposed to 100 μL of 0.5% DMSO (vehicle control), culture medium without any test compound (blank control), AC, MA and HA (in different concentrations), respectively. Following exposure to DDAs (24, 48 and 72 h), the medium was replaced by 50 μL MTT for each well. After additional incubating for 4 h, the formazan crystals were dissolved in DMSO and the absorbance of each well was measured at 570 nm on an ELISA reader.

### Assessment of cell morphology

The morphologies of rat myocardial cells H9c2 after exposure to 6.9 × 10^−8^ M AC, 10.5 × 10^−8^ M HA or 11.8 × 10^−8^ M MA for 24 h were examined under a contrast microscope (OlympusBX51, Japan).

### Detection of apoptosis by flow cytometric analysis

After treated with AC (2.5 × 10^−8^ M), MA (5.0 × 10^−8^ M) or HA (4.0 × 10^−8^ M) for 24 h, H9c2 cells were harvested and washed twice with cold PBS. Then the cells were stained with PI and annexin V-fluorescein isothiocyanate (FITC) for 15min in the dark at room temperature. Cellular fluorescence was measured by flow cytometry analysis with Beckman Coulter FC-500 type flow cytometer (Beckman Coulter, Miami, USA).

### Absorption spectra of AC-DNA, HA-DNA and MA-DNA adducts

After exposure to AC (12.0 × 10^−8^ M), HA (14.0 × 10^−8^ M) or MA (14.0 × 10^−8^ M) for 24 h, rat myocardial cells H9c2 were harvested and washed with DMEM. DNA adducts were extracted and measured using a TU-1901 spectrophotometer (Beijing Purkinje General Instrument Co., Ltd, China). UV absorption spectra for extracted DNA of cells from vehicle control and blank control were also recorded.

### Absorption spectra of DDAs interaction with CT-DNA

0.4 mL of 2 mM CT-DNA, 1 mL of 0.1 mM NR and 4 milliliter of Tris-HCl buffer, were mixed into a 10 mL test tube. Different concentrations of DDAs (AC: 4.0~16.0 × 10^−5^ M, MA: 5.0~35.0 × 10^−5^ M, HA: 5.0~35.0 × 10^−5^ M) were added into the solution after 5 min stabilization. Diluted the mixture to 10 mL with deionized water. After 10 min, the UV absorption spectra of the complex were measured with the wavelength range from 200 to 700 nm, and the slit width was 2 nm. The blanks corresponding to the buffer were subtracted to correct the absorbance at room temperature.

### Fluorescence quenching studies

In order to determine the optimal molar ratio of probe and CT-DNA, the fluorescence intensity of 0.1 μM NR with varying the concentrations of CT-DNA from 0 μM to 2 μM were measured. Then, the solution, containing a certain concentration of NR-DNA complex ([DNA]/[NR] = 8, the fluorescence intensity of NR dye could not increase with sequentially adding DNA into NR solution), was titrated by successive addition of DDAs (AC: 4.0~16.0 × 10^−5^ M, MA: 5.0~35.0 × 10^−5^ M, HA: 5.0~35.0 × 10^−5^ M). Fluorescence spectra of the complex were recorded after 5 min stabilization at three temperatures (298, 304, and 310 K). The excitation wavelength was at 546 nm, and the emission spectra were in range of 560 nm–750 nm. All slits width was fixed at 10 nm. The fluorescence intensities were carried out at room temperature with a F-7000 spectrofluorimeter (Hitachi, Japan).

### Melting studies

Thermal denaturation experiments were carried out by monitoring the absorbance intensities of the CT-DNA with or without DDAs at different temperatures. The absorbance at 259 nm were plotted as a function of the temperature ranging from 20 °C to 100 °C. The melting temperature (Tm) of DNA and DNA adduct were estimated using the equation *f*_*ss*_ = (*A - A*_*0*_)/(*A*_*f*_ *- A*_*0*_), where A_0_ is the absorbance at 20 °C and A is the absorbance at 100 °C. The curves were generated based on *f*_*ss*_ versus temperature (*T*)^39^.

### Effects of ionic strength

Sodium chloride was used to study the influence of ionic strength on the fluorescence intensity of the DDA-NR-DNA systems. DNA-NR and DDA-NR-DNA solutions fixed with NaCl at concentrations of 0.1 M, 0.2 M and 0.3 M were prepared respectively, and fluorescence intensities were measured.

### Statistical analysis

SPSS 16.0 statistical software package was carried out for statistical analysis. Multiple comparisons were performed by one-way ANOVA, following by the SNK test for differences between groups. The significance was tested as *p* < 0.05.
