# Author Correction: Cytotoxicity of *Aconitum* alkaloid and its interaction with calf thymus DNA by multi-spectroscopic techniques

**DOI:** 10.1038/s41598-018-30986-6

**Published:** 2018-08-17

**Authors:** Fei Liu, Xiaoxin Tan, Xu Han, Xiang Li, Nan Li, Weijun Kang

**Affiliations:** 1 School of Basic Medical, Hebei University of Chinese Medicine, Shijiazhuang, Hebei Province China; 2 School of Public Health, Hebei Medical University, Shijiazhuang, Hebei Province China; 3 Institute of Viral Disease, Hebei Center for Disease Control and Prevention, Shijiazhuang, Hebei Province China

Correction to: *Scientific Reports* 10.1038/s41598-017-15240-9, published online 06 November 2017

In the original version of this Article, Fei Liu was incorrectly listed as one of the corresponding authors. The correct corresponding author for this Article is Weijun Kang. Correspondence and request for materials should be addressed to: kangwj@hebmu.edu.cn. This error has now been corrected in the HTML and PDF versions of the Article.

